# Mortality in the Emergency Department and the Effectiveness of Conventional Safety Event Reporting

**DOI:** 10.7759/cureus.45472

**Published:** 2023-09-18

**Authors:** Nancy Jacobson, Abigail Miller, Sean A Mackman, Anshul Bhatnagar, Jamie Aranda, Matthew Chinn, Ronny Otero

**Affiliations:** 1 Emergency Medicine, Medical College of Wisconsin, Milwaukee, USA; 2 Emergency Medicine, Baylor College of Medicine, Houston, USA

**Keywords:** safety event reporting, patient mortality review, morbidity and mortality, patient safety improvement, quality improvement and patient safety

## Abstract

Background

Patient mortality reviews identify care, system, and process deficiencies. Patient deaths undergo quarterly review in our academic emergency department (ED), whereas in other departments, mortality reviews are requested by the pronouncing physician within 24 hours. In the ED, individual physicians encounter barriers to 24-hour reviews, including feasibility, the perception of futility, re-exposure to traumatic events, and a high frequency of pre-hospital and non-preventable deaths. This quality review aimed to determine the preventable death rate, contributing factors to ED patient mortality, cases requiring further review, and the capture rate of individual case submissions into the patient safety reporting system.

Methods

A retrospective chart review was performed on all patient deaths occurring in our ED from July 2019 to February 2020. All patients 18 years or older who were pronounced dead in the ED during our data collection period were included. Patients declared deceased pre-hospital, on an inpatient floor, or in the operating room were excluded. Deaths were assessed for characteristics such as sex, presence of a pulse upon arrival, diagnostics and interventions performed, and whether the cause of death was traumatic or medical. Deaths were categorized on a 5-point Likert scale ranging from "not preventable" to "likely preventable." The presence or absence of contributing factors and the need for further review were recorded.

Results

Of the 166 reviewed cases, 87% (n=144) were non-preventable due to a terminal condition upon arrival, 12% (n=20) were non-preventable despite maximal efforts, 0.6% (n=1) were non-preventable despite a medical or systems error, and 0.6% (n=1) were possibly preventable due to a medical or systems error. No cases were definitively preventable. Only 1.2% (n=2) of cases required further safety review. In 55% (n=91) of cases, the patient arrived without a pulse. Medical deaths (60%, n=100) outnumbered traumatic deaths (39%, n=64). The most utilized diagnostic test was ultrasound (67%, n=111), and the most utilized intervention was advanced cardiac life support (59%, n=98).

Conclusion

There is a high prevalence of unpreventable deaths in the ED (99%, n=164). Only two cases (1.2%) were identified for further patient safety review. Standard safety event reporting practices correctly identified all possibly preventable ED deaths.

## Introduction

Reducing patient mortality is a universal objective for frontline physicians, administrative leaders, and healthcare systems. As a clinical outcome that is highly visible, easily measurable, and particularly undesirable, mortality often serves as a key measure of healthcare quality [1]. Therefore, patient mortality reviews have been proposed as a potential mechanism to identify critical deficiencies in healthcare systems and processes [1]. Previous research has shown that implementing department- and institution-wide mortality reviews can reveal systematic quality gaps and likely lead to improvements in clinical care [1-6].

Compared to physicians in other specialties, emergency physicians may face unique challenges in implementing systematic patient mortality reviews. These challenges stem from the relatively higher frequency of pre-hospital and non-preventable deaths. Other potential barriers include a lack of feasibility, perceptions of futility, and re-exposure to traumatic events for the care team. Despite these obstacles, deaths occurring in emergency departments (EDs) require review to identify patient safety cases, emerging trends, and system-based barriers to quality care. Furthermore, departments of emergency medicine may obtain relatively greater benefits from patient mortality reviews, as prior research indicates that 51% of ED deaths are preventable [7]. Another study concludes that while preventable trauma deaths have decreased with the onset of formal trauma programs and case reviews, preventable patient mortality still exists to a significant degree in the ED setting [8]. Consequently, implementing ED patient mortality reviews could lead to substantial improvements in quality and reductions in mortality, compared to those seen in other clinical settings.

In our hospital, two workflows exist for systematic mortality review. Our Department of Emergency Medicine (EM) conducts a quarterly review that consists of EM faculty and senior residents evaluating cases triggered by quality measures, including ED patient death (hereafter referred to as "EM quarterly review"). Meanwhile, physicians caring for patients in the inpatient hospital setting are prompted to submit a mortality review form within 24 hours of having declared a patient deceased (hereafter referred to as "real-time review"). Regardless of the clinical practice environment, any care team member can submit concerning cases for peer review via the electronic patient safety event reporting platform (hereafter referred to as "individual case reporting").

We conducted a retrospective observational review of ED deaths with the aim of better describing the clinical characteristics of patient mortality in the ED, determining the preventable death rate, analyzing contributing factors to ED patient mortality, identifying cases requiring further review, and enumerating any events not detected by individual event reporting and/or EM quarterly review.

This work was previously presented as a poster at the 2022 Society of Academic Emergency Medicine in May 2022; the 2022 Wisconsin Chapter - American College of Emergency Physicians in April 2022; and the Society of Academic Emergency Medicine Great Plains Regional Meeting in September 2022.

## Materials and methods

Setting and Patient Selection

This is an observational retrospective chart review of patient mortality in an adult academic ED at a Level 1 trauma center. We identified cases using our Electronic Health Record (EHR) system's built-in patient chart analysis functionality. We included all adult patient deaths in our ED between July 2019 and February 2020 as a convenience sample. Exclusion criteria were patients under 18 years of age and patients whose deaths occurred outside the ED (e.g., pre-hospital, inpatient, or operating room settings). Approval for this study was obtained from the Quality Improvement/Quality Assessment Review Committee of the Department of Emergency Medicine at the Medical College of Wisconsin and was IRB-exempt.

Review Process

Charts were manually reviewed by two authors (NJ and AM), and author consensus was used to determine the ultimate conclusions of preventability during the chart review. The authors were blinded as to whether a case had undergone departmental peer review or any quarterly review findings. Reviewing authors included the Department of Emergency Medicine Director of Quality, Safety, and Experience, and a senior resident participating in an Emergency Medicine Clinical Administration and Operations Resident Elective. The reviewing authors were trained in quality and safety review processes, in accordance with the recommendations of the Agency for Healthcare Research and Quality and in keeping with the tenets of just culture [9]. The study team met frequently throughout the review period to monitor the reviewing authors' determinations and ensure consistency in review. A five-point scale, ranging from "not preventable" to "likely preventable," was used to record the presence or absence of contributing factors [10-11]. Reviewing authors also determined the need for further review. These methods were developed in accordance with recommended practices for chart review in Emergency Medicine research, as described by Gilbert et al. [12].

Data were collected, including patient sex, presence of a pulse upon arrival, diagnostics and interventions performed, and the cause of death (traumatic, medical, or unclear). The authors additionally evaluated three primary aspects of each case using a confidential, HIPAA-compliant, novel rating tool that mimicked the prompts used for inpatient real-time reviews at our institution. First, they assessed the preventability of patient death on a 5-point scale ranging from "not preventable" to "likely preventable" [10-11]. Second, they characterized the presence or absence of contributing factors to patient death to assess the overall quality of care and identify potential opportunities for improvements in seven selected domains of clinical care (Table 1). Third, the need for further peer review was determined.

**Table 1:** Factors for Identifying Quality Gaps Across Health Care Domains Reviewers were asked to identify potential opportunities for improvements in seven select domains of clinical care.

Domains of Care	Potential Areas of Improvement
Patient Evaluation	History of present illness or physical exam
Decision-Making	Differential diagnosis, treatment plan, diagnostic plan, diagnostic conclusion
Diagnosis/Treatment Ordering	Orders, order delivery, inappropriate protocol or treatment, work-up
Diagnosis/Treatment Performance	Procedure preparation, unexpected complication, procedure performance, treatment choice
Patient Monitoring	Complication response, monitoring methods, monitoring frequency, follow-up
Documenting	Legible records, timeliness, and communication of patient assessment
Communication and Coordination	Timely response to requests for help, communication with patient/family, coordination of care, communication with the medical team.

Data Analysis

Standard statistical measures and methods were used for analysis. Frequencies and percentages were calculated for categorical variables. The percentages of preventable and non-preventable deaths and cases where clinical errors and/or systems issues occurred were calculated. Sub-analyses of specific categories of potential systems opportunities were also conducted. Microsoft Excel, version 2016 (Microsoft Corporation, Redmond, Washington, United States) was used for all analyses.

## Results

Mortality Characteristics

A total of 166 patient deaths were reviewed. The key characteristics of these patient cases are summarized in Table 2. Most patients were male (66.3%; n=110), while the remaining 56 individuals (33.7%) were female. Seventy-one patients (42.8%) were pulseless on arrival to the ED, and 91 individuals (54.8%) presented with a pulse. Electronic medical record (EMR) documentation was unclear as to whether or not the remaining four individuals (2.4%) had a pulse on arrival. One hundred patients (60.2%) presented with a medical emergency, while 64 (38.6%) had a traumatic emergency; two individuals (1.2%) had an unclear form of a life-threatening emergency.

**Table 2:** Descriptive Statistics of Patient Deaths Reviewers recorded data including patient sex, if the patient had pulses on arrival to the ED, and if the etiology of death was medical, traumatic, or unclear.

Characteristic	Total (n=166)
Patient sex	
Female	56 (33.7%)
Male	110 (66.3%)
Pulse on arrival?	
Yes	71 (42.8%)
No	91 (54.8%)
Uncertain	4 (2.4%)
Classification of emergency	
Medical	100 (60.2%)
Traumatic	64 (38.6%)
Uncertain	2 (1.2%)

**Figure 1:**
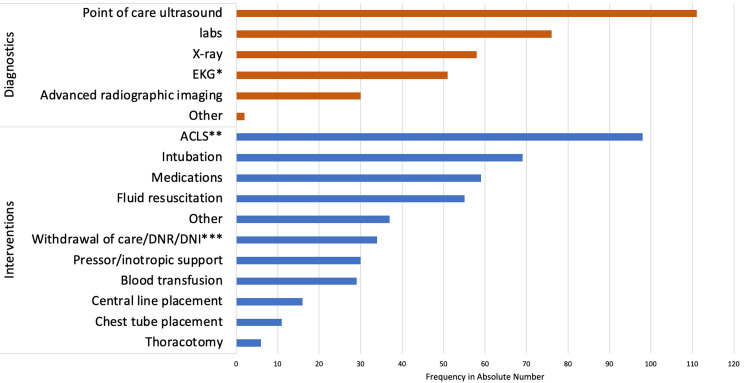
Frequency of Diagnostic Testing and Interventions Among Patient Deaths For patients who became deceased in the emergency department, the most utilized diagnostic test was point of care ultrasound, and the most common interventions were ACLS and intubation. *EKG = electrocardiogram. **ACLS = advanced cardiac life support. ***DNR = do-not-resuscitate, DNI = do-not-intubate.

The utilization of various diagnostic tests and interventions in cases of patient death is summarized in Figure 1. The three most commonly utilized diagnostic tests were point-of-care ultrasound (66.9%, n=111), STAT laboratory testing (45.8%, n=76), and x-ray imaging (34.9%; n=58 ). An electrocardiogram (EKG) was ordered for 51 individuals (30.7%) and advanced radiographic imaging was obtained for 30 patients (18.1%). The three most frequently utilized interventions were advanced cardiac life support (ACLS) (59.0%, n=98), intubation (41.6%, n=69), and any medication (35.5%, n=59). Fluid resuscitation was another commonly utilized intervention and was ordered for 55 individuals (33.1%). All other interventions, including continuous inotropic support, blood transfusion, and central line placement, were used in <25% of all cases. Withdrawal of care, due to a do-not-resuscitate (DNR), do-not-intubate order (DNI), or medical futility, occurred in 34 individuals (20.5%).

Mortality Review

**Figure 2:**
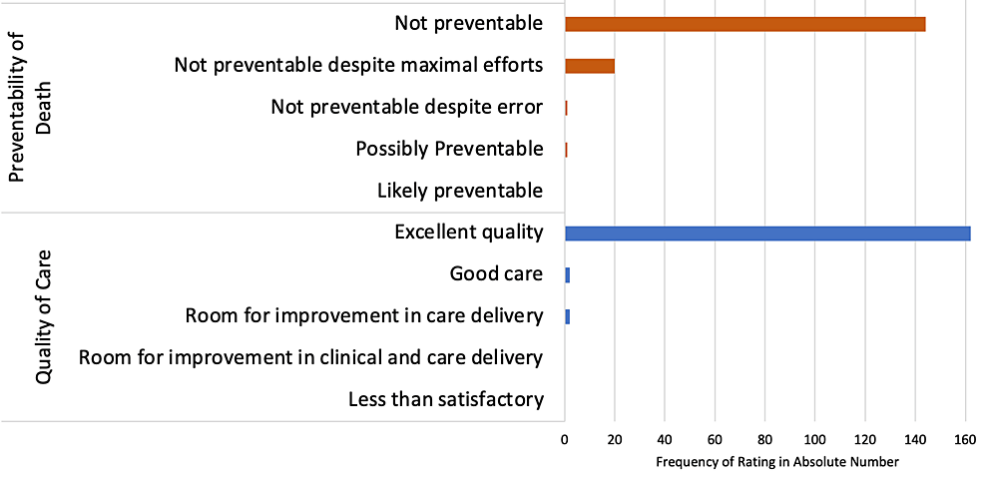
Preventability of Patient Death and Quality of Care Reviewers rated 144 cases (86.7%) of death as non-preventable. In 20 cases (12.0%), patient death was non-preventable despite maximal treatment efforts. In one case (0.6%), patient death was also considered non-preventable despite a medical or systems error and one case (0.6%) was possibly preventable death due to a medical issue or systems flaw. Quality of care was excellent in 162 cases (97.6%). Reviewers found room for improvement due to errors in care delivery systems in two cases (1.2%).

The assessed preventability of patient death and quality of care is summarized in Figure 2. In 144 cases (86.7%), reviewers believed patient death was non-preventable due to a terminal illness or condition upon arrival at our ED. In 20 additional cases (12.0%), patient death was deemed non-preventable despite maximal treatment efforts by ED personnel. In one case (0.6%), patient death was also considered non-preventable despite a medical or systems error. Lastly, one individual (0.6%) was deemed to have a possibly preventable death due to a medical issue or system flaw. No patient was found to have a definitively preventable death.

Quality of care was judged as excellent in 162 cases (97.6%). In two patients (1.2%), aspects of clinical care could have been improved; however, the quality of care was subsequently deemed to represent good care. In two cases (1.2%), moderate room for improvement was found due to errors in care delivery systems. Reviewers did not identify any cases where there was room for improvement due to errors in both care and clinical delivery systems.

Specific areas of improvement were noted at least once in six of the seven studied care domains. The only domain of care in which no issue was observed was diagnosis and treatment performance. Inadequate HPI, incomplete differential diagnosis, inappropriate protocol or treatment, improper monitoring frequency and methods, poor communication of patient assessment, and substandard coordination of care each occurred once during the study period. Reviewers found that 164 cases (98.8%) did not require additional quality review; however, one patient death (0.6%) was recommended for departmental review and another (0.6%) for institutional review.

## Discussion

Patient safety incidents are often underreported, obscuring serious quality issues and leading to poor patient outcomes [13]. Methods like systematic mortality reviews-such as real-time mortality reviews and review methodologies akin to EM quarterly reviews-have been proposed as ways to discover previously missed safety events [7]. However, our retrospective observational ED mortality review did not uncover any previously unreported safety incidents. Individual case reporting accurately identified all ED patient deaths that resulted from substandard care or system-based barriers (1.2%, n=2). Early identification through individual case reporting facilitated in-depth analysis of significant quality gaps within our institution. As institutions adopt resource-intensive real-time or quarterly mortality reviews, it is crucial to weigh their cost-to-benefit ratio. It may be more effective to invest in fostering a culture of safety and facilitating safety event reporting, which in our experience was 100% effective in identifying cases that required peer review.

Published literature offers conflicting perspectives on the benefits and drawbacks of systematic mortality reviews. The identification of unique quality gaps is a significant advantage of successful patient mortality reviews [1-6]. These can lead to the formulation of new guidelines and policies that tackle identified quality gaps. For instance, statewide maternal mortality reviews in Illinois led to revised guidelines and new legislation that improved hemorrhage management for delivering mothers [5]. Moreover, systematic physician feedback, which could be encouraged by departmental or institutional reviews, can result in small yet crucial improvements in clinical outcomes and practice [14]. Nonetheless, systematic reviews might meet resistance from physicians and could undermine a culture of safety. If mortality reviews negatively impact a culture of safety, the review systems themselves should be scrutinized for effectiveness and necessity. In a radiology department considering the adoption of a systematic review process, 67% of physicians believed that systematic reviews would damage professional relationships, and 42% feared harm to their job security [15]. The re-exposure to particularly distressing events may also lead to "second victim syndrome" [16]. Given the increasing awareness of physician wellness and burnout, the psychological and social effects of implementing a systematic mortality review process must be considered.

Our review found that an overwhelming majority of patient deaths in our department were unpreventable. Despite potential errors or maximal treatments, approximately 99% of all patient deaths in our setting could not have been averted. We assessed healthcare quality to be excellent in most cases. These findings contrast with prior research, showing considerably higher proportions of preventable ED deaths [7,17]. Differences between our study and earlier work may arise from variations in patient populations, institutional factors, and pre-hospital EMS and treatment protocols. Our study included all types of ED deaths, such as traumatic arrests, withdrawal of care, and patients who arrived pulseless, whereas earlier research excluded these subsets [7]. Notably, a large number of patients who died in our ED arrived without a pulse. This likely contributed to the high percentage of deaths deemed non-preventable in our review. This variability underscores the need for emergency medicine departments to understand their unique clinical circumstances and identify the factors driving patient mortality within their institutions. Our findings suggest that retrospective mortality reviews can serve as tools for identifying trends in mortality characteristics like pulselessness upon arrival or the traumatic versus medical etiology of death. However, more traditional case reporting methods can also be equally effective at identifying cases requiring further departmental and institutional scrutiny.

Limitations

In this retrospective review, cases were assessed by physicians from within the department, as opposed to external physicians. This arrangement may introduce cognitive biases, as reviewers might be unconsciously inclined to rate a case as "not preventable" or to conclude that the standard of care was met. Further, the involvement of senior resident physicians in the review team could result in an inherent power differential. This might make resident reviewers hesitant to critique the care provided by attending physicians. While these potential limitations exist, they are mitigated by our methodology, which includes author consensus with the department's Director of Quality and Safety and the use of a validated 5-point scale. Although a scale was employed, the case evaluations remain inherently subjective due to diverse practice techniques in complex cases. However, the nature of quality reviews is subjective, both in this study and in clinical practice. Thus, subjectivity remains an omnipresent limitation in retrospective reviews, whether they are conducted in real-time, quarterly, for individually submitted cases, or in studies like this one.

Our ED is an adult academic ED at a Level 1 trauma center. This setting may skew the patient population toward those suffering from penetrating trauma or severe critical illnesses. As a result, we observed higher proportions of patients presenting without a pulse and higher proportions of cases rated as not preventable, either due to terminal conditions upon arrival or despite maximal efforts. While this context somewhat limits the generalizability of our findings, the data remain applicable to other academic EDs, many of which are tertiary care and trauma centers. Additionally, when viewed alongside other published literature, our review effectively illustrates the variability in the proportions of preventable deaths across different EDs. This emphasizes the importance of characterizing patient presentations during mortality reviews and of interpreting mortality rates and review outcomes within the specific clinical context of each ED.

## Conclusions

In conclusion, we successfully conducted a retrospective quality assessment of mortality in an urban academic ED. We recognized that many of our patients arrive without a pulse, that we frequently use POC ultrasound as a diagnostic test in patients who die in the ED, and that our most frequent intervention is ACLS. Our review uncovered a high prevalence of unpreventable death in this setting but did not uncover any patient safety incident that was previously missed by individual case reporting. As institutions continue to implement mortality reviews, ED physicians should consider whether quality gaps and patient safety events can be effectively identified through currently existing protocols.
